# Nutrition Facts in Multiple Sclerosis

**DOI:** 10.1177/1759091414568185

**Published:** 2015-02-09

**Authors:** Paolo Riccio, Rocco Rossano

**Affiliations:** 1 Department of Sciences, University of Basilicata, Potenza, Italy

**Keywords:** complementary alternative medicine, gut microbiota, inflammation, lifestyle, multiple sclerosis, nutrition

## Abstract

The question whether dietary habits and lifestyle have influence on the course of multiple sclerosis (MS) is still a matter of debate, and at present, MS therapy is not associated with any information on diet and lifestyle. Here we show that dietary factors and lifestyle may exacerbate or ameliorate MS symptoms by modulating the inflammatory status of the disease both in relapsing-remitting MS and in primary-progressive MS. This is achieved by controlling both the metabolic and inflammatory pathways in the human cell and the composition of commensal gut microbiota. What increases inflammation are hypercaloric Western-style diets, characterized by high salt, animal fat, red meat, sugar-sweetened drinks, fried food, low fiber, and lack of physical exercise. The persistence of this type of diet upregulates the metabolism of human cells toward biosynthetic pathways including those of proinflammatory molecules and also leads to a dysbiotic gut microbiota, alteration of intestinal immunity, and low-grade systemic inflammation. Conversely, exercise and low-calorie diets based on the assumption of vegetables, fruit, legumes, fish, prebiotics, and probiotics act on nuclear receptors and enzymes that upregulate oxidative metabolism, downregulate the synthesis of proinflammatory molecules, and restore or maintain a healthy symbiotic gut microbiota. Now that we know the molecular mechanisms by which dietary factors and exercise affect the inflammatory status in MS, we can expect that a nutritional intervention with anti-inflammatory food and dietary supplements can alleviate possible side effects of immune-modulatory drugs and the symptoms of chronic fatigue syndrome and thus favor patient wellness.

Multiple sclerosis (MS) is a chronic, inflammatory, and autoimmune disease of the central nervous system (CNS), leading to widespread focal degradation of the myelin sheath, variable axonal and neuronal injury, and disabilities in young adults, mostly women. The disease is characterized by disseminated and heterogeneous perivascular inflammatory processes at the blood–brain barrier (BBB), with involvement of autoreactive T cells, B lymphocytes, macrophages, and microglial cells against brain and spinal cord white matter (McFarland and Martin, 2007; Constantinescu and Gran, 2010; Kutzelnigg and Lassmann, 2014).

Antibodies (Krumbholz et al., 2012), activated complement (Ingram et al., 2014), cytokines, mitochondrial dysfunction (Su et al., 2009), reactive oxygen species (ROS; Gilgun-Sherki et al., 2004), and matrix metalloproteinases (MMPs; Liuzzi et al., 2002; Rossano et al., 2014) may cooperate to yield the pathology.

From the clinical point of view, there are at least two main forms of the disease: the relapsing-remitting MS (RRMS; about 85% of clinical cases) and the primary-progressive MS (PPMS; about 15% of the clinical cases) (Dutta and Trapp, 2014; Lublin et al., 2014). In RRMS, which usually evolves in secondary-progressive MS (SPMS), relapses are associated with increased systemic inflammation and formation of lesions in the brain, followed by more or less complete remissions, whereas the pathogenesis of PPMS is characterized by progressive neurological damages rather than relapses and remissions.

At present, there are at least 10 disease-modifying therapies that have been found to slow disease progression and prevent some disability symptoms, but only in the case of RRMS. However, as the disease is complex in nature and unique in the individual course, no patient responds to therapy in the same way (Loleit et al., 2014). Similarly, there are no truly reliable biomarkers that allow for everyone to evaluate the effectiveness of treatment and it is therefore important to discover novel markers of the disease (Fernandez et al., 2014).

The lack of response to immune-modulatory therapies in the case of PPMS, otherwise effective in the treatment of RRMS, may be due to different pathogenic mechanisms acting in RRMS and PPMS. However, this is not true with regard to inflammation: A significant association between inflammation and neurodegeneration has been observed in the brain not only in acute and relapsing MS but also in the secondary and primary progressive MS (Frischer et al., 2009; Lassmann, 2013), and active MS lesions are always associated with inflammation (Kutzelnigg and Lassmann, 2014). Thus, inflammation must be the target for the treatment of both forms of the disease.

## Linking Inflammation with Dietary Habits and Lifestyle

What causes the inflammatory processes in MS? MS is a complex disease, and the genetic and the immunological components are not sufficient to explain its origin. Actually, MS has a multifactorial nature and various environmental factors or metabolic conditions may have a role in its development (Ascherio, 2013): viral infections (Ascherio et al., 2012; Venkatesan and Johnson, 2014), heavy metal poisoning (Latronico et al., 2013; Zanella and Roberti di Sarsina, 2013), smoking (Jafari and Hintzen, 2011), childhood obesity (Munger, 2013), low vitamin D status (Ascherio et al., 2014), or incorrect lifestyle, including wrong dietary habits (Riccio, 2011; Riccio et al., 2011; Riccio and Rossano, 2013).

None of the above-mentioned environmental factors alone can explain the disease; however, the following considerations make more attractive the involvement in MS of dietary habits and lifestyle, rather than infections or smoking, as factors that may influence the course of the disease:

- *Geographical distribution:* MS is more prevalent in Western countries with the highest income and most distant of the equator. Features of these countries are sedentary lifestyle, high-calorie diet rich in saturated fats of animal origin (Western diet), and low sunshine exposure (WHO and MSIF, 2008).
- *Effect of migration:* With the migration from an area of high incidence of MS to another place with low incidence before age of 15 years, the low risk is acquired, while the migration after this age does not change the level of risk. This aspect may be linked with nutritional, rather than with infectious or toxicological environmental factors (McLeod et al., 2011).
- *Low availability of vitamin D:* Another environmental factor related to diet and geographical distribution is the availability of vitamin D, which is lower at latitudes with lower exposure to sunlight. Patients with MS have a low content of vitamin D (Ascherio et al., 2014), but this is true also for other chronic inflammatory diseases (Yin and Agrawal, 2014).
- *Postprandial inflammation:* High animal fat/high sugar and refined carbohydrate diet is associated with postprandial inflammation (Erridge et al., 2007; Ghanim et al., 2009; Margioris, 2009).
- *High body mass index:* High body mass index (BMI) before age 20 is associated with 2× increased risk (Hedström et al., 2012). Note that BMI is correlated with gut microbiota status.
- *Similarity with other inflammatory diseases related to wrong dietary habits*: MS has some similarities with inflammatory bowel disease (IBD; Cantorna, 2012): both have low vitamin D and are influenced from environmental factors (Dam et al., 2013). Furthermore, glatiramer acetate (GA, or Copolymer 1/Copaxone) is beneficial in both diseases (Aharoni, 2013) and there is increased incidence of IBD among MS patients.

## How Food Affects the Course of Inflammatory Diseases: A Basic Approach

The observations reported above suggest that the nutritional status may influence the course of MS. However, the question arises of how dietary molecules could exacerbate or ameliorate MS symptoms, and in general how they could favor or downregulate inflammation at molecular level. In particular, it is important to clarify what are the targets of dietary molecules and the molecular mechanisms involved, if any.

**Figure 1.**
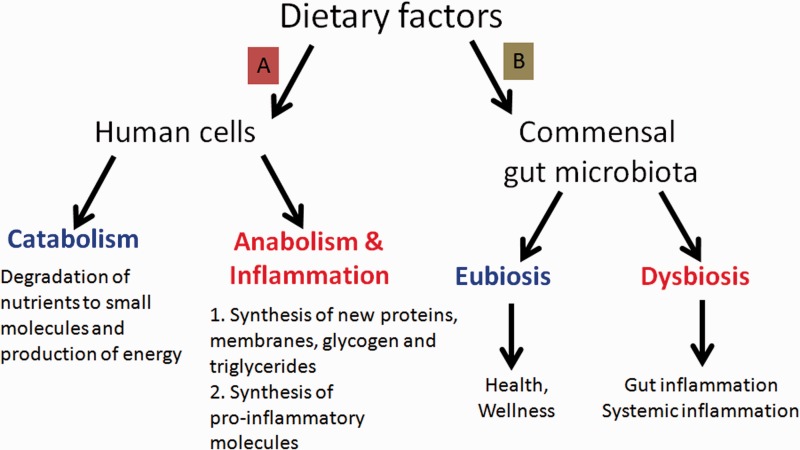
The two routes by which diet can influence our health: (A) the metabolism of our cells and (B) the population of our gut microbiota.

Fundamentally, we can say that the food we consume has a broad impact on our development, behavior, health condition, and lifespan by acting on two main targets: (A) the cells of our body and (B) the commensal gut microbiota (Figure 1).

- On one hand, different kind and amount of dietary factors can interact with enzymes, transcription factors, and nuclear receptors of human cells. This may induce specific modifications of cellular metabolism toward either catabolism or anabolism and modulate the inflammatory and autoimmune responses in our body (Desvergne et al., 2006).
- On the other hand, we have to consider the impact of diet and lifestyle on our intestinal microflora. We are indeed metaorganisms living with trillions (10^14^) of microbial cells (roughly 10 times the cells of our body) and thousands of different microorganisms known as the *gut microbiota*. This complex ecosystem is an essential part of our organism and influences both our immune system and our metabolism. Therefore, it has a strong impact on our health.

In health, there is a close mutualistic and symbiotic relationship between gut microbiota and humans, and gut microbiota provides a number of useful metabolic functions, protects against enteropathogens, and contributes to normal immune functions. This is the normal state of the human intestinal microbiota, called *eubiosis*. Distortion from eubiosis, linked with a decrease of intestinal biodiversity and increase of pathogenic bacteria, is called *dysbiosis*. The most common consequence of a dysbiotic gut microbiota is the alteration of the mucosal immune system and the rise of inflammatory, immune, metabolic, or degenerative diseases (Chassaing and Gewirtz, 2014).

Different kinds and amounts of dietary factors elicit the selection of specific gut microbial populations changing type and number of microbial species toward eubiosis or dysbiosis, simply acting through the preferential feeding of one or the other microbial population. If our diet favors the change to a dysbiotic gut microbiota, this may lead to gut inflammation, alteration of intestinal immunity, and then to systemic inflammation and chronic inflammatory diseases.

## How Dietary Factors Influence the Metabolism of Human Cells and Modulate Inflammation

To understand how dietary molecules can directly influence the metabolism of human cells, it is necessary to describe first what are the enzymes and transcription factors involved in catabolism or anabolism in the cell.

**Figure 2.**
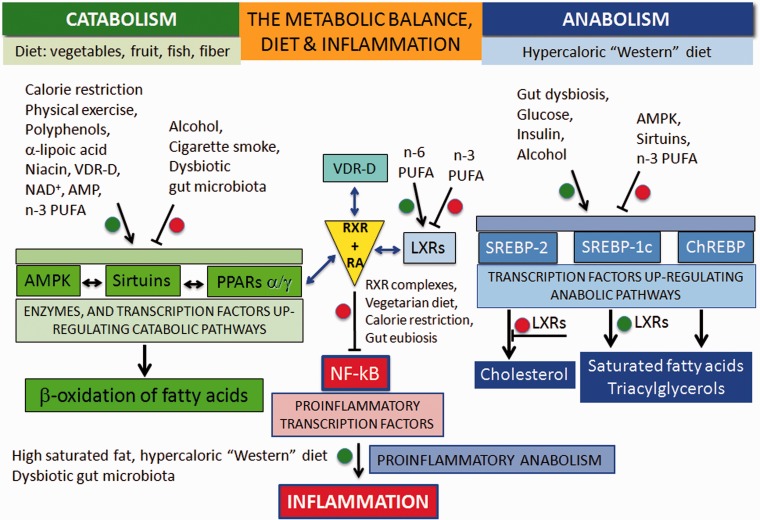
A schematic and simplified representation of how natural dietary factors can direct cell metabolism toward oxidative metabolism (on the left), biosynthesis (on the right), and NF-kB-induced inflammation (at the bottom of the figure, in red) by their binding to nuclear receptors, transcription factors, and enzymes having transactivating properties. Intermediate receptors and response elements are not indicated. *Note.* The complete list of agonists (+) and antagonists (−), including some common drugs, of the enzymes, nuclear receptors, and transcription factors, is shown below: • AMPK: (+) calorie restriction, physical exercise, AMP, Ghrelin, alfa-lipoic acid, adiponectin, flavonoids, nonflavonoids, metformin, salicylate; (−) dysbiotic gut microbiota; leptin. • Sirtuins: (+) VDR-D, calorie restriction, alfa-lipoic acid, resveratrol, niacin, TRP, NAD^+^; (−) alcohol, cigarette smoke, nicotinamide. • PPARs alfa/gamma: (+), polyphenols, Sirtuins; coffee component hydroxyl hydroquinone (HHQ), thiazolidinediones, cannabinoid agonists, 15d PGJ2; ibuprofen, statins. • NF-kB: (+) Saturated and *trans* fatty acids, oncoproteins, ROS, TNF-α, IL-1 b, LPS, viral infections; (−) calorie restriction, polyphenols, n-3 PUFA, butyrate, Sirtuins. • LXRs: (+) n-6 PUFA, oxysterols; glucose; (−) n-3 PUFA. • SREBP-1c: (+) LXRs, gut dysbiosis, alcohol, insulin; (−) n-3 PUFA, metformin, Sirtuins, AMPK • SREBP-2: (−) LXRs, statins • ChREBP (+) glucose; (−) LXRs, statins. PPAR = peroxisome proliferator-activated receptor; LXR = liver X receptor; RXR = retinoid X-receptor; NF-kB = nuclear transcription factor-kB; SREBP = steroid regulatory element-binding protein; ChREBP = carbohydrate responsive element-binding protein; Sirtuins = SIRT-1/2, deacetylating enzymes; AMPK = AMP-activated protein kinase; n-3 PUFA = omega-3 polyunsaturated fatty acids.

As shown on the left in Figure 2, oxidative metabolism is upregulated by two enzymes and a nuclear receptor. The enzymes are the AMP-activated protein kinase (AMPK; Steinberg and Kemp, 2009) and the Sirtuins (SIRT), a group of histone deacylating enzymes, which are activated by NAD^+^ (Zhang et al., 2011; Rice et al., 2012). The nuclear receptor is represented by the isotypes of the peroxisome proliferator-activated receptors (PPARs; Desvergne and Wahli, 1999; Burns and VandenHeuvel, 2007).

PPAR isotypes upregulate the transcription of genes involved in the beta-oxidation of fatty acids in mitochondria and peroxisomes and form a network with AMPK and Sirtuins pathways. The AMPK-Sirtuins-PPAR pathway is activated by a lifestyle based on calorie restriction and physical exercise, as well as by some bioactive molecules (polyphenols, found in vegetables and fruits, and omega-3 (n-3) long-chain polyunsaturated fatty acids [PUFA], found in fish). Ligand-activated PPAR isotypes form heterodimeric complexes with the retinoid X-receptor (RXR), which, in turn, is activated by 9-cis-retinoic acid (RA).

Conversely, as shown on the right in Figure 2—like on the other dish of an imaginary balance—high intake of energy-dense nutrients leads to the upregulation of anabolism, including lipogenesis and cell growth, through the activation of the sterol regulatory element-binding proteins, SREBP-1c and SREBP-2 (Xu et al., 2013), and the carbohydrate responsive element-binding protein, ChREBP (Xu et al., 2013). SREBP-1c and SREBP-2 are under the control of the nuclear receptors called the *liver X receptors* (LXR; Mitro et al., 2007; Nelissen et al., 2012). LXR isotypes, which are activated by the cholesterol derivatives oxysterols and glucose, have a relevant role in the synthesis of lipids by activating SREBP-1c and the synthesis of triacylglycerols, while inhibiting SREBP-2 and the synthesis of cholesterol.

Central to the understanding of the link between diet and inflammation are two transcription factors involved in inflammation and autoimmunity: the nuclear transcription factor-kB (NF-kB) and the activator protein (AP-1; Yan and Greer, 2008). In MS, both NF-kB and AP-1 are activated and induce the expression of several proinflammatory genes and the production of proinflammatory molecules. The cause of their activation in MS is not known but, as shown in Figure 2 for NF-kB, this can be activated not only by viruses, cytokines, and oxidative stress but also by some dietary components such as saturated fatty acids or *trans* unsaturated fatty acids, which therefore can be considered proinflammatory.

Downregulation of the proinflammatory NF-kB can be achieved by the inhibitory binding of the RA-activated forms of the retinoid X-receptor isotypes (RXRs; Pérez et al., 2012; Zhao et al., 2012; Fragoso et al., 2014).

**Figure 3.**
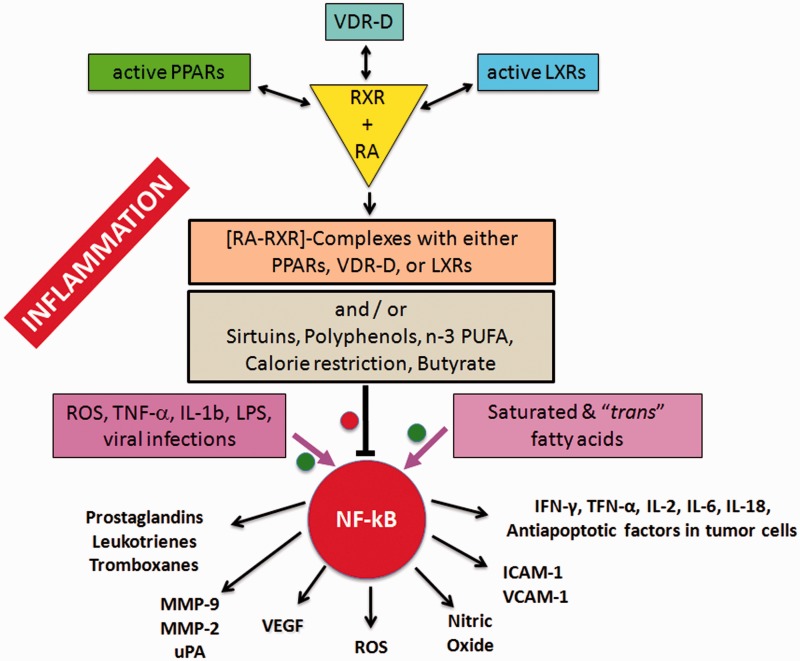
Portion of Figure 2 showing in detail the relationship between diet and inflammation, at the example of the proinflammatory transcription factor NF-kB. *Note.* The production of some proinflammatory molecules is also shown. MMP = metalloproteinase; VEGF = vascular endothelial growth factor; ROS = reactive oxygen species; ICAM-1 = intercellular adhesion molecule; VCAM-1 = vascular cell adhesion molecule.

As shown in the center of Figure 2 and more in detail in Figure 3, the active forms of RA-RXRs are heterodimers resulting from their association with specific ligand-activated nuclear receptors, namely PPARs, LXRs, and vitamin D receptor (VDR).

All three nuclear receptors—PPAR, LXR, and VDR—must be activated by specific ligands. As indicated in Figure 2, the ligands can be specific dietary factors and this clarify how cells respond to changes in nutritional status and regulate energy homeostasis but represents also the molecular key to understanding how nutrients can influence the course of chronic inflammatory diseases (Heneka et al., 2007; Zhang-Gandhi and Drew, 2007; Krishnan and Feldman, 2010; Cui et al., 2011; Schnegg and Robbins, 2011; Gray et al., 2012).

Therefore, each of the three nuclear receptors—PPAR, LXR, and VDR—competes for the binding to RA-RXR and forms hetero-complexes that can inhibit NF-kB and exert a tight control over the expression of inflammatory genes, thus integrating metabolic and inflammatory signaling. It is clear that there is competition between the three receptors PPAR, LXR, and VDR-D, for the binding with RA-RXR, but this competition should have an influence only on metabolism and not on inflammation, because it is not yet known which of the three heterodimers is more effective in inhibiting NF-kB.

**Figure 4.**
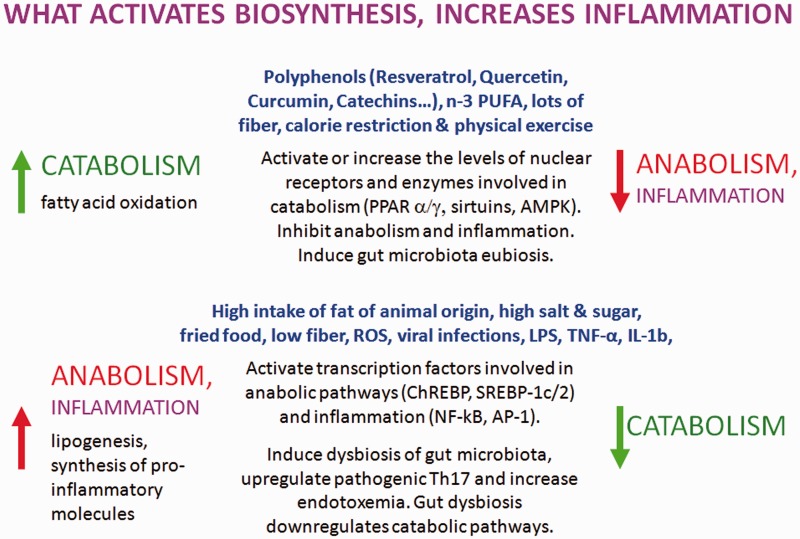
Summary of the relationship between dietary habits, lifestyle, and metabolic balance. The figure emphasizes how, as in a two-dish balance, the molecules that on one hand promote oxidative metabolism and on the other hand downregulate the biosynthetic pathways, including, in particular, the proinflammatory ones. Conversely, the figure shows how Western eating habits and lifestyle have the opposite effect and promote biosynthesis, including the production of proinflammatory molecules.

Obviously, the production of proinflammatory molecules in the course of relapses is a biosynthetic process: It is sustained by hypercaloric diets and counteracted by low-calorie diets. In principle, what favors anabolism will promote the inflammatory processes, while what favors catabolism will contrast them (Figure 4).

## How Dietary Factors Influence Composition and Biodiversity of Gut Microbiota and Alter Host–Microbiota Relationship

### The Link Between Lifestyle, Dietary Habits, and Gut Microbiota Composition

The composition of the intestinal microflora is highly individual and is influenced by many factors such as diet, physical activity, stress, medications, age, and so forth. Each of us has a unique set of at least 100 to 150 species of bacteria.

An easy way to discuss about the effect of food and lifestyle on gut microflora is to restrict the overview to only two dominant bacterial divisions—the *Bacteroidetes* and the *Firmicutes*—accounting for about 90% of the total, as it has been shown that the ratio *Bacteroidetes/Firmicutes* (B/F) is influenced by long-term dietary habits (Cani and Delzenne, 2009; Wu et al., 2011; Lozupone et al., 2012; Tremaroli and Bäckhed, 2012; Panda et al., 2014).

A comparative study of De Filippo et al. (2010) in children from Florence and from Burkina Faso in Africa showed that long-term dietary habits have significant effects on human gut microbiota.

In this study, the Burkina Faso diet was based on the consumption of plant polysaccharides such as millet and sorghum (10 g fibers/day and 662–992 kcal/day), whereas the diet of Italian children was Western style, based on proteins, animal fat, sugar-sweetened drinks, and refined carbohydrates (5.6 g fibers/day and 1,068–1,512 kcal/day). Analysis of fecal samples in the children from Africa showed the prevalence of the *Bacteroidetes* (73%)—mainly *Prevotella* and *Xylanibacter*—and low levels of *Firmicutes* (12%). On the contrary, a prevalence of *Firmicutes* (51%) over the *Bacteroidetes* (27%) was observed in Italian children, but the *Bacteroidetes* shifted from *Prevotella* and *Xylanibacter* to *Bacteroides*. These latter are usually selected among the *Bacteroidetes* because they can use also simple sugars in addition to complex glycans, and simple sugars are normal components of Western diets.

In conclusion, the B/F ratio increases in association with a diet rich in complex carbohydrates (nondigestible by our enzymes) because the symbiotic and usually nonharmful *Bacteroidetes*, such as *Prevotella* and *Xylani bacter*, love to have complex glycans to eat. Bacteria consuming complex glycans produce butyrate, which downregulate the activation of proinflammatory NF-kB (Figure 3).

Conversely, Western, energy-dense diets change the gut microbiota profile and increase the population of *Firmicutes* (including the *Mollicutes*), more suited to extract and harvest energy, but often pathogenic (Moschen et al., 2012).

### The Link Between Dysbiotic Gut Microbiota and Chronic Inflammation

**Figure 5.**
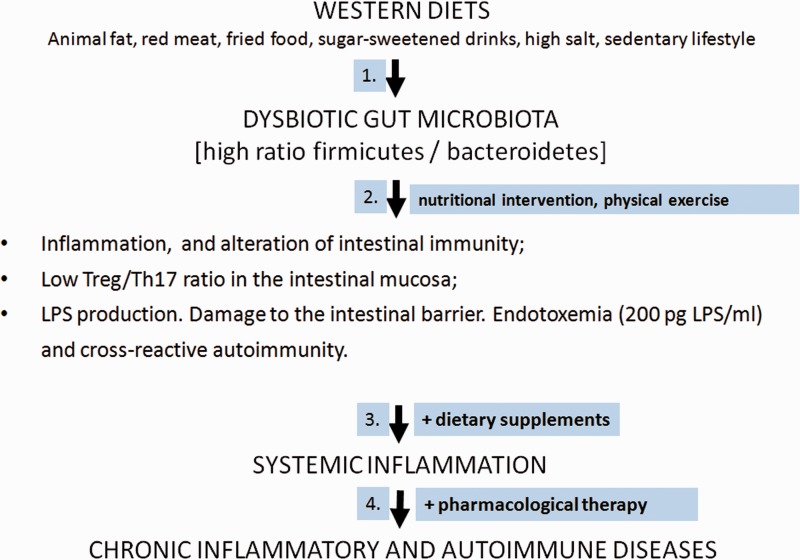
The sequence of events representing the influence of low-fiber, hypercaloric, Western diets on the development of gut dysbiosis, intestinal and systemic inflammation, and the subsequent appearance of chronic inflammatory diseases. The figure shows the stages in which it is suggested to apply in sequence nutritional intervention, dietary supplements, and therapy.

In a dysbiotic gut microbiota, the B/F ratio is low and the possibly pathogenic *Firmicutes* prevail over *Bacteroidetes* (Figure 5). The failure of microbial balance and the decrease of biodiversity occurring in dysbiosis lead to the disruption of the complex interplay between the microbiota and its host and contribute to low-grade endotossemia, and chronic intestinal and systemic inflammation. With the onset of systemic inflammation, the risk of chronic inflammatory and immune-mediated diseases increases (Tilg et al., 2009; Brown et al., 2012; Maynard et al., 2012).

Actually, in the presence of a dysbiotic microbiota, gut endotoxin/lipopolysaccharide (LPS) is increased, regulatory T cells (Treg) are defective, and the aryl hydrocarbon receptors and proinflammatory Th17 cells are activated (Cani et al., 2008; Veldhoen et al., 2008).

LPS leads to the dysfunction of the mucosal barrier and affects other tissues when its plasma level increases above 200 pg/ml serum. The increased gut permeability due to the dysbiotic gut microbiota may be exemplified by the passage of IgA and IgG antibodies against gluten and gliadin, also observed in MS patients (Reichelt and Jensen, 2004).

### The Link Between Dysbiotic Gut Microbiota and MS

In our previous work, we have proposed that the model linking microbiota alteration—due to Western diet and lifestyle—and the failure of the correct communication between the microbiota and the intestine, leading to low-grade endotoxemia and systemic autoimmune inflammation, might be valid also for the pathogenesis of MS (Fernández et al., 2012; Riccio, 2011). In fact, MS shares with other chronic inflammatory diseases common mechanisms, all probably based on the persistence of low-grade endotoxemia related to wrong lifestyle and dietary habits together with a latent dysbiosis. Moreover, the existence of a gut microbiota-brain axis, which is now more than an emerging concept, suggests that intervention on gut microbiota may be a fruitful strategy for future treatment of complex CNS disorders (Cryan and Dinan, 2012).

The possible direct link between gut microbiota and MS has been shown experimentally by Berer et al. (2011). Using transgenic mice, Berer et al. have shown that gut commensal bacteria can trigger a relapsing-remitting autoimmune disease driven by myelin-specific CD4+ T cells and demyelination, given the availability of MOG—the autoantigen myelin oligodendrocyte glycoprotein. In another study, it was shown that antibiotic treatment directed to alter gut microflora suppresses experimental allergic encephalomyelitis (EAE; Yokote et al., 2008).

These findings suggest that gut microbiota may play a crucial role in the starting phase of MS and may also predispose host susceptibility to other CNS autoimmune diseases as well as to neuropsychiatric disorders such as autism, depression, anxiety, and stress. A new concept of gut microbiota-brain axis is emerging (Wang and Kasper, 2014).

On these grounds, understanding the role of gut microbiota in health and disease can lay the foundation to treat chronic diseases by modifying the composition of gut microbiota through the choice of a correct lifestyle, including dietary habits. Moreover, direct manipulation of the gut microbiota may improve adaptive immune response and reduce inflammatory secretions. For example, because a specific role of intestinal Th17 cells has been suggested in MS immunopathology (Sie et al., 2014), promoting Treg cell differentiation and reducing pathogenic Th17 cells might prevent recurrence of autoimmunity in MS patients (Issazadeh-Navikas et al., 2012).

On these grounds, the discovery that the defect of the Treg/Th17 balance observed in MS models is also present in MS patients, could have important clinical implications, as this defect can be modulated by changes in the microbiota composition, which in turn is modulated by dietary changes (David et al., 2014).

## Proinflammatory Dietary Factors

The components of the diet whose intake must be controlled to avoid the rise of inflammatory processes in MS, as well as in other chronic inflammatory diseases, are as follows:

1. Saturated fatty acids of animal origin;
2. Unsaturated fatty acids in the *trans* configuration (hydrogenated fatty acids);
3. Red meat;
4. Sweetened drinks, and in general hypercaloric diets rich in refined (low-fiber) carbohydrates, in addition to animal fat;
5. Increased dietary salt intake;
6. Cow’s milk proteins of the milk fat globule membrane (MFGM proteins).

## Fat of Animal Origin

Saturated fatty acids of animal origin, which are found in foods such as whole milk, butter, cheese, meat, and sausages, are the components of the diet taken into account more frequently for their deleterious influence on the course of MS.

In 1950, Swank suggested that the consumption of saturated animal fat is directly correlated with frequency of MS, but a link between restricted intake of animal fat and remission of MS was reported only in 2003 (Swank and Goodwin, 2003). According to Swank and Goodwin, high-fat diets lead to the synthesis of storage lipids and cholesterol and cause a decrease of membrane fluidity and possible obstruction of capillaries, and the onset or increase of inflammation.

Other more recent studies indicate that the action of saturated fat is controlled at the transcriptional level and influence both gene expression, cell metabolism, development, and differentiation of cells. More in general, the assumption of animal fat is often linked to a high-calorie intake, which is on its own a detrimental factor for many chronic inflammatory diseases. Finally, as described later in this article, an excess of saturated animal fat leads to a dysbiotic intestinal microbiota, dysfunction of intestinal immunity, and low-grade systemic inflammation and represents a possible cause of some human chronic disorders.

### Trans Fatty Acids

*Trans* fatty acids (TFAs) are unsaturated fatty acids that contain at least one nonconjugated double bond in the *trans* configuration (Bhardwaj et al., 2011).

As products of partial hydrogenation of vegetable oils, they were introduced in the 1960s to replace animal fat, but only much later it was found that they have the same deleterious effect on the metabolism and, as the saturated fatty acids, increase the levels of cholesterol and promote the formation of abdominal fat and weight gain. TFAs intake was found to be positively associated with gut inflammation and the upregulation of proinflammatory citokines in Th17 cell polarization (Okada et al., 2013). Moreover, TFAs interfere with the metabolism of natural unsaturated fatty acids, which have the cis configuration.

TFAs are found in margarine and other treated (hydrogenated) vegetal fat, in meat and dietary products from ruminants and in snacks. They may be present also in French fries and other fried food, as they are also formed in the frying.

### Red Meat

Red meat contains more iron heme than white meat. The iron is easily nitrosylated and this facilitates the formation of endogenous nitroso-compounds (NOCs; Joosen et al., 2010). Red meat intake shows indeed a dose–response relation with NOCs formation, whereas there is no such relation for white meat. NOCs are mutagenic: induce nitrosylation and DNA damage. Processed (nitrite-preserved) red meat increases the risk. Heterocyclic amines are formed during cooking of meat at high temperatures, but this is not specific for red meat (Joosen et al., 2010).

Abnormal iron deposits have been found at the sites of inflammation in MS (Williams et al., 2012) and consumption of red meat is associated with higher levels of γ-GT and hs-CRP (Montonen et al., 2013).

Noteworthy, we do not have N-glycolylneuraminic acid (Neu5Gc), a major sialic acid, because an inactivating mutation in the CMAH gene eliminated its expression in humans. Metabolic incorporation of Neu5Gc from dietary sources—particularly red meat and milk products—can create problems, as humans have circulating anti-Neu5Gc antibodies and this implies the possible association with chronic inflammation (Padler-Karavani et al., 2008).

Finally, meat contains arachidonic acid (the omega-6 (n-6) PUFA, which is the precursor of proinflammatory eicosanoids [prostaglandins, thromboxanes, and leukotrienes]) and activates the Th17 pathway (Stenson, 2014).

### High Intake of Sugar and Low Intake of Fiber

The high intake of sugar-sweetened beverages and refined cereals, with low fiber content, increases rapidly the number of calories and glucose level. The subsequent increase of insulin production upregulates the biosynthetic pathways and *inter alia* the production of arachidonic acid and its proinflammatory derivatives.

### Increased Dietary Salt Intake

Increased dietary salt intake might be an environmental risk factor for the development of autoimmune diseases, as it has been found that it can induce pathogenic Th17 cells and related proinflammatory cytokines in EAE (Kleinewietfeld et al., 2013; Wu et al., 2013). Th17 cells have been involved in the development of MS.

### Cow’s Milk Fat and the Proteins of the Milk Fat Globule Membrane

Milk fat is dispersed in a homogeneous way and protected from oxidation, thanks to a membrane made of lipids and particular proteins called *proteins of the milk fat globule membrane* (MFGM; Riccio, 2004). These proteins, which account for only 1% of milk proteins, have an informational rather than a nutritional value. In human lactation, they are needed for the correct formation of the digestive, nervous, and immune systems in infants. This flow of information is obviously not relevant, or not required at all, in adulthood and, as well, in the case of cow’s milk taken for human nutrition. In adult age, MFGM proteins of cow’s milk no longer have an informational role and may be eliminated from the diet together with milk fat.

The removal of MFGM proteins from whole cow’s milk is particularly relevant in the case of MS. The most representative MFGM protein (40% of total MFGM proteins), butyrophilin (BTN), is indeed suspected to have a role in MS, as it is very similar to MOG, one of the candidate autoantigen in MS. BTN and MOG share the same behavior in MS experimental models, and MOG/BTN cross-reactive antibodies have been found in MS, in autism and in coronary heart disease (CHD; Riccio, 2004). On these grounds, the patient with MS should avoid the intake of whole cow’s milk and prefer skimmed milk, which, in addition, has no animal fat.

Another point of view is that of Swanson et al. (2013). They have found that BTN or BTN-like molecules might have a regulatory role in immunity and therefore they suggest that BTN or BTN-like molecules could be useful to induce Treg development.

### Hypercaloric Diets and Postprandial Inflammation

After each meal, we may experience a transient and moderate oxidative stress and a moderate inflammatory response depending on type and quantity of food. Dietary habits based on a frequent and persistent exposure to meals with high intake of salt/animal fat and *trans* fat/sugar-sweetened drinks stresses our immune/metabolic system and the subsequent possible failure of homeostasis may lead to immune and metabolic disorders of diverse nature.

Taken together, the diet-dependent stress might be due to following reasons: (a) calorie intake: the higher the calories, the more the oxidative stress induced; (b) glycemic load of a meal: acute postprandial glycemic peaks may induce a release of insulin much higher than necessary; (c) lipid pattern: saturated animal fat, *trans* fatty acids, and omega-6 (n-6) long-chain PUFA promote postprandial inflammation. As reported in the following sections, postprandial inflammation is attenuated or suppressed by n-3 PUFA and polyphenols, calorie restriction, and physical exercise.

## Anti-Inflammatory Natural Bioactive Compounds: Useful to Tackle MS and Prevent Relapses?

Specific bioactive dietary molecules are able to counteract the effects of pathogenic microbial agents and downregulate the expression of inflammatory molecules. Among them, the most important compounds are the polyphenols and carotenoids from vegetables, n-3 PUFA from fish, vitamins D and A, thiol compounds such as lipoic acid, and oligoelements such as selenium and magnesium.

Most of the above-mentioned compounds, with exception of PUFA, which are not antioxidant, are known for their antioxidant properties. The rationale for the use of antioxidants in MS is based on the observation that oxidative stress is one of the most important components of the inflammatory process leading to degradation of myelin and axonal damage. However, it is now known that dietary antioxidants have additional biological properties going far beyond the simple antioxidant activity. Indeed, they are able to counteract the negative effects of microbial agents and saturated or *trans* fatty acids, downregulating the expression of proinflammatory molecules, oxidative stress, and angiogenesis.

### Polyphenols

All polyphenols—which are present in vegetables, cereals, legumes, spices, herbs, fruits, wine, fruit juices, tea, and coffee—have anti-inflammatory, immune-modulatory, anti-angiogenic, and antiviral properties and stimulate the catabolic pathways (Gupta et al., 2014; Wang et al., 2014). They are found in plants in the form of glycosides, esters, or polymers, too large to enter the intestinal membrane. Aglycons released from gut microbiota are conjugated to glucuronides and sulfates in intestine and liver. Their solubility and bioavailability are very poor (µM; Visioli et al., 2011).

From a structural point of view, polyphenols include flavonoids and nonflavonoids molecules (Bravo, 1998). The most important flavonoids are quercetin (onions, apples, citrus fruit, and wine; Min et al., 2007; Sternberg et al., 2008), catechins (green tea; Friedman, 2007), and daidzein and genistein (soy; Castro et al., 2013; Zhou et al., 2014). The most important nonflavonoids are resveratrol (chocolate, peanuts, berries, black grapes, and red wine; Das and Das, 2007; Cheng et al., 2009; Shakibaei et al., 2009), curcumin (spice turmeric of ginger family, curry; Prasad et al., 2014), and hydroxytyrosol (olive oil; Hu et al., 2014).

It has been found that the anti-inflammatory effect of polyphenols *in vitro* may depend on their chemical structure (Liuzzi et al., 2011). Thus, a mixture of flavonoids and nonflavonoids may be more effective than supplementation with only one polyphenol.

Two examples of the most studied polyphenols are quercetin and resveratrol. Quercetin is present mainly as a glucoside. Most of its effects are additive to those of interferon-β. Quercetin is not toxic, but its oxidation product, quercetin quinone, is very reactive toward the SH groups of proteins and glutathione and may be toxic (Boots et al., 2008). Addition of lipoic acid or N-acetylcysteine can limit the toxic effects.

Resveratrol is glucuronated in the liver and absorbed in this form mainly in the duodenum but only in very limited amount. Depending on its concentration, resveratrol can induce the death of a wide variety of cells by necrosis or apoptosis. In this regard, it is commonly accepted that resveratrol has neuroprotective effects; however, it has been also reported that it can exacerbate experimental MS-like diseases (Sato et al., 2013). These discrepancies can be attributed to the different concentrations used *in vitro* or bioavailable *in vivo*, as resveratrol has opposite effects at concentrations of 10^−5 ^M (proliferation of human mesenchimal cells) and 10^−4 ^M (inhibition of proliferation). In our experience, resveratrol has a neurotrophic effect on cortical neurons in culture only at very low concentration, whereas at higher concentration, it may have toxic effect. But in the case of oxidative stress, resveratrol has neuroprotective properties also at the higher concentrations.

### Vitamin D, Vitamin A, Carotenoids, Other Vitamins, and Oligoelements

Other compounds and elements that may be useful as supplements in MS are the vitamins D, A, E, C, B12 (Mastronardi et al., 2004), and niacin (Penberthy and Tsunoda, 2009), and oligoelements such as selenium (Boosalis, 2008) and magnesium (Galland, 2010).

Vitamin D has immune-modulatory roles and represents the most promising dietary molecule for the treatment of chronic inflammatory diseases such as MS (Smolders et al., 2008; Pierrot-Deseilligny, 2009; Cantorna, 2012; Ascherio et al., 2014). As already mentioned, it is generally believed that the special geographical distribution of MS in the world can also be attributed to the reduced availability of vitamin D_3_, due to insufficient exposure to sunlight in some countries, and the lack of active vitamin D may be another possible cause of environmental origin of MS. However, low levels of active vitamin D may be due also to its altered metabolism or function not only to the exposure to sunlight. In fact, the failure of vitamin D_3_ (cholecalciferol) supplementation to show beneficial effects on body weight or on the course of inflammatory diseases may be due to the persistence of its deficiency despite its administration.

Vitamin D_3_ (cholecalciferol), formed after exposure to sunshine, is hydroxylated in the liver to 25-(OH) D_3_ (calcidiol) by the P450 enzymes CYP27A1 or CYP2R1, and subsequently activated in the kidney by CYP27B1 to 1α, 25-(OH)_2_ D_3_ (calcitriol). This latter, the active form of vitamin D, is inactivated by CYP24A1 to 1α, 24,25-(OH)_3_ D_3_ (calcitroic acid). This means that the levels of active vitamin D depend on the relative rates of its synthesis via CYP27B1 and its modifications via CYP24A1 (Schuster, 2011). High CYP24A1 expression, induced by endogenous compounds and xenobiotics, might lead to low levels of vitamin D and cause or enhance chronic inflammatory diseases and cancer. On these grounds, it is important to follow up the level of vitamin D in the course of vitamin D administration. If vitamin D levels remain low, the expression of CYP24A1 mRNA should be examined, and determination of CYP27B1 and CYP24A1 activities and their inhibition should be tested (Chiellini et al., 2012, Kósa et al., 2013).

Another important aspect regards the VDR. The active metabolite of vitamin D—1α, 25-dihydroxyvitamin D—binds to VDR, and the complex VDR-D controls the expression of several genes involved in processes of potential relevance to chronic diseases. As represented in Figures 2 and 3, the VDR-D complex competes with ligand-activated PPARs or LXRs for the binding to RA-RXR. The heterodimeric complexes bind to the proinflammatory transcription factor NFkB and downregulate the synthesis of proinflammatory molecules. In this context, when evaluating the effectiveness of vitamin D supplementation in the course of MS, one should consider the eventual polymorphisms affecting the VDR, which has been recently associated with obesity, inflammation, and alterations of gut permeability (Al-Daghri et al., 2014).

Moreover, the finding that that VDR-D activate the Sirtuin SIRT-1 (An et al., 2010; Polidoro et al., 2013) suggests that vitamin D has an influence also on cell metabolism and therefore may have properties similar to those of many other natural dietary supplements: upregulate oxidative metabolism and downregulate inflammation.

Finally, it should be considered that there are differences between data in humans and experimental models. Actually, in humans, unlike in mice, obesity is associated with poor vitamin D status (Bouillon et al., 2014).

Among the carotenoids, the most important is lycopene (tomato, water melon, and pink grape fruit; Rao and Rao, 2007). Besides to be a very strong antioxidant, lycopene can give beta-carotene and retinoic acid, and the latter can activate the RXR receptor (Figure 2). Although higher intakes of dietary carotenoids, vitamin C, and vitamin E did not reduce the risk of MS in women (Zhang et al., 2001), the relevance of lycopene and vitamin A against inflammation cannot be disregarded.

### Omega-3 (n-3) Essential Fatty Acids and Poly-Unsaturated Fatty Acids from Vegetables, Seafood, and Fish Oil

n-3 essential fatty acids (EFA) and PUFA represent a valid alternative to saturated fatty acids of animal origin.

Vegetable and vegetable oils contain the essential fatty acids linoleic acid (n-6) and linolenic acid (n-3). n-6 and n-3 fatty acids have opposite effects and their presence in the diet should be equivalent (Schmitz and Ecker, 2008). However, in Western diets, the ratio n-6/n-3 is increased from 6 to 15 times and this leads to a higher incidence of cardiovascular and inflammatory diseases. In fact, the linoleic acid leads to the formation of arachidonic acid (20:4), the precursor of the proinflammatory eicosanoids prostaglandins-2, leukotrienes-4, and thromboxanes-2. The synthesis of these eicosanoids is favored by insulin, and inhibited by aspirin, as well as by the n-3 long-chain PUFA EPA (eicosapentaenoic acid) and DHA (docosahexaenoic acid), which derive from n-3 linolenic acid.

Both DHA and EPA are found in seafood and fish oil. Both show remarkable anti-inflammatory, anti-thrombotic, and immune-modulatory activities, comparable with those of statins (Calder, 2006; Farooqui et al., 2007). n-3 PUFA inhibit inflammatory processes and the synthesis of fatty acids and cholesterol, and instead they stimulate the oxidation of fatty acids. On this basis, in chronic inflammatory diseases such as MS, n-3 essential fatty acids (EFA) and n-3 PUFA should prevail in the diet over the n-6 fatty acids. It is interesting to note that DHA is present in high concentrations in the brain and its levels decrease in patients with MS.

In cultured microglial cells activated by LPS, fish oil is as effective as interferon-β in inhibiting the expression of MMP-9 (gelatinase B), an important mediator of neuro-inflammation (Liuzzi et al., 2004, 2007). Moreover, n-3 PUFA significantly decreased MMP-9 levels in few clinical trials, indicating that n-3 PUFA may represent a good complementary treatment in the course of MS (Weinstock-Guttman et al., 2005; Mehta et al., 2009; Shinto et al., 2009). Fish oil has been also found to improve motor performances in healthy rat pups (Coluccia et al., 2009).

n-3 PUFA act in synergy with aspirin on AMPK and COX enzymes but with different mechanisms. Noteworthy, in the presence of aspirin, EPA and DHA form new anti-inflammatory bioactive molecules called resolvins, protectins, and maresins, which are able to reduce cellular inflammation and inflammatory pain (Xu et al., 2010; Hong and Lu, 2013; Serhan and Chiang, 2013). This may be a relevant aspect related to the nutritional intervention in MS. Indeed, the inflammatory processes associated to MS could be also due to the low ratio omega-3 (anti-inflammatory)/omega 6 (inflammatory) PUFA and thereby to the low production of adequate amounts of resolution-inducing molecules lipoxins, resolvins, and protectins that suppress inflammation. Hence, administration of omega-3 PUFA together with aspirin or directly of lipoxins, resolvins, and protectins may form a new approach in the prevention and treatment of MS and other neuroinflammatory diseases. Furthermore, other anti-inflammatory and antiangiogenic eicosanoids can also be produced by the P450 CYP enzymes from EPA and DHA (Yanai et al., 2014). In this context, it should be taken into consideration that statins may interfere negatively with the metabolism of n-3 and n-6, as they can decrease the n-3/n-6 ratio. Thus, treatment with statins should be associated with n-3 PUFA supplementation (Harris et al., 2004).

Seeds oils, from sunflower, corn, soybean, and sesame, contain more n-6 fatty acids than n-3 fatty acids and therefore their assumption should be limited in MS, in order to limit the level of proinflammatory eicosanoid production. On the other hand, coconut oil has a high content of saturated fatty acids. Among vegetable oils, olive oil should be preferred for the good ratio between saturated and unsaturated fatty acids, and because it contains the antioxidant hydroxytyrosol.

### Thiolic compounds as Dietary Supplements

Compounds containing thiol groups (–SH) such as α-lipoic acid (ALA), glutathione, and N-acetylcysteine (NAC) should be taken into consideration as possible dietary supplements to be used for the complementary treatment of MS.

As polyphenols, ALA (Salinthone et al., 2008; green plants and animal foods) has immunomodulatory and anti-inflammatory properties. ALA stabilizes the integrity of the BBB and stimulates the production of cAMP and the activity of protein kinase A. Also NAC might be useful in neurological disorders. It passes through the BBB and protects from inflammation (Bavarsad Shahripour et al., 2014).

## The Mediterranean Diet

A recent systematic review and meta-analysis of intervention trials provide evidence that Mediterranean diet patterns reduce inflammation and cardiovascular mortality risk and improves endothelial functions (Schwingshackl and Hoffmann, 2014). These findings are as much encouraging as you think that the true Mediterranean diet is a little different from the one currently described.

It is generally agreed that the Mediterranean diet is based on consumption of extra-virgin olive oil, unrefined cereals, legumes, diverse vegetables (in particular tomatoes) and fruits, dairy products (mostly as pecorino cheese, ricotta, mozzarella, and yogurt), fish and fishery products, and low consumption of animal fat and meat. However, currently, the Mediterranean diet tends to a high consumption of pasta and bread, which means a high intake of gluten.

Once, in true Mediterranean diet, in Southern Italy, meat was eaten two or at most three times a week, only olive oil was used for cooking (extra-virgin quality and the most possible raw), but notably the intake of gluten was about half compared with the current intake. The pasta was eaten with the classic home-made tomato sauce, but in alternative, it was most often mixed with other gluten-free foods. The most common recipes were pasta and potatoes; pasta with either green beans, or artichokes, zucchini, eggplant, turnips, or cabbage; pasta with a mix of vegetables and legumes (minestrone: vegetable soup); and pasta with chickpeas, beans, or lentils. The sugar-sweetened drinks of today were not known. A high assumption of gluten-rich food may lead to nonceliac asymptomatic gluten sensitivity, mucosal intestinal damage, changes in gut microbiota, and low-grade intestinal inflammation. In conclusion, the Mediterranean diet is good, but the intake of gluten must be limited and must be whole grains.

## Inflammatory and Anti-Inflammatory Lifestyle

### Smoking (Proinflammatory)

Only a few studies have been carried out on the impact of smoking on the course of MS and results are conflicting, perhaps because its effects are difficult to ascertain and enucleate from other factors. Weiland et al. (2014) have found no association between smoking and relapse rate or disease activity, but do not exclude that smokers might have a significantly lower health-related quality of life than non-smokers, whereas Manouchehrinia et al. (2013) found that smoking is associated with more severe disease.

However, as it is shown in Figure 2, it can be expected that cigarette smoke may worsen the course of MS, as it may inhibit the anti-inflammatory activity of Sirtuins (Caito et al., 2010). The oxidative and carbonyl stress induced by cigarette smoke can be reversed by resveratrol (Liu et al., 2014).

### Alcohol Consumption (Proinflammatory)

Recent studies shows that alcohol (beer, wine, or liquor) consumption is not associated to MS risk (Massa et al., 2013; Hedström et al., 2014). However, as also shown in Figure 2, alcohol may inhibit the Sirtuin SIRT1 and activate the transcriptional activity of SREBP-1c (You et al., 2008), thus promoting the biosynthesis of lipids and inflammation at the expense of oxidative metabolism.

There are other two aspects of ethanol that should be considered. First, the metabolism of ethanol converts a large number of NAD^+^ molecules to NADH, limiting the availability of NAD^+^ required for the activity of Sirtuins. Second, as a substrate of the P450 enzymes, ethanol can interfere with the metabolism of drugs, which are transformed by the same enzymes. The result may be the prolongation and the enhancement of drug action. Altogether, alcohol should be considered as a molecule that interferes with the normal metabolism and facilitates the inflammatory process, complicating the possibility of improving the wellbeing of the patient.

### Calorie Restriction (Anti-Inflammatory)

High-calorie intake and a meal rich in refined carbohydrates and sugar increase insulin level and favors biosynthesis, including the production of proinflammatory molecules and the production of free radicals. Calorie restriction, obtained by decreasing food intake or by intermittent fasting (one day and the other not), upregulates the level of SIRT1 (Zhang et al., 2011), increases the level of AMP and upregulates AMPK, increases adiponectin levels and upregulate or activate its receptors (Lee and Kwak, 2014), and downregulates oxidative damage, lymphocyte activation, and the progression of experimental models of MS (Piccio et al., 2008, 2013). The effects of calorie restriction can be mimicked by agonists (resveratrol and other polyphenols), acting on the same targets (SIRT1, AMPK).

### Physical Exercise (Anti-Inflammatory)

Physical exercise is now an almost accepted practice also for MS patients and is commonly applied in order to decrease the symptoms of chronic fatigue and prevent or slow the onset of disability. However, the importance of physical exercise goes beyond that of simple muscle activity and should be rather considered in a holistic context in which diet, exercise, therapy, and social interchange, all play a role for the wellness of MS patients (Gacias and Casaccia, 2013).

Dietary control and exercise practice have been proposed by the WHO (2010) to attenuate or prevent human chronic diseases.

From a molecular point of view, physical exercise exerts its beneficial effect by acting on the protein kinase AMPK axis and the AMPK–Sirtuins–PPAR-δ network, upregulating oxidative metabolism and downregulating biosynthetic pathways and inflammation (Narkar et al., 2008). As AMPK has a key role in energy balance, it is important to mention its agonists. Resveratrol and AMPK agonists such as metformin, a drug used in type 2 diabetes, can mimic or enhance the effect of physical activity and are effective in experimental encephalitis (Nath et al., 2009).

Physical exercise influences the quality of life and may stimulate the production of anti-inflammatory cytokines (Florindo, 2014). Furthermore, physical exercise lowers plasma levels of leptin and reduces gene expression of leptin receptors in the liver (Yasari et al., 2009), while increasing adiponectin levels and adiponectin receptors activity (Lee and Kwak, 2014).

The association of physical exercise with calorie restriction leads to a significant reduction of inflammatory markers (Reed et al., 2010).

Recent studies carried on adult C57BL/6 J male mice have shown that exercise stimulate brain mitochondrial activity, potentiate neuroplasticity, and is associated to mood improvement, as it decrease anxiety-like behaviors in the open field and exert antidepressant-like effects in the tail suspension test (Aguiar et al., 2014). Other studies performed on rats showed that exercise can alter the composition and diversity of gut bacteria (Petriz et al., 2014).

On these grounds, MS patients should practice mild physical exercise (brisk walking, swimming, or even dancing), if possible in the course of a rehabilitation program.

## Nutritional Clinical Trials in MS So Far

Unfortunately, nutritional clinical trials in MS are only very few. Some of them were based on diets low in saturated fat, either without supplements (Swank and Goodwin, 2003) or with omega-3 fat supplements (Nordvik et al., 2000; Weinstock-Guttman et al., 2005). Other clinical trials were based on the administration of single dietary supplements only: either vitamin D, or fish oil (n-3 PUFA), or lipoic acid. Clinical trials with single polyphenols were performed only in cancer. Dietary supplements have never been used together and have never been associated with dietary prescription.

Taken together, clinical attempts to clarify the role of nutrition in MS were considered only promising of poor quality or with no clear results (Farinotti et al., 2007, 2012). In particular, as reported by Farinotti et al. in their Cochrane review (2012), supplements such as n-3 PUFA seem to have no major effect on the main clinical outcome in MS, but they may reduce the frequency of relapses over 2 years. Data available were considered to be insufficient or of uncertain quality to assess a real effect from PUFA supplementation. In some studies, slight possible benefits in relapse outcomes were found with omega-6 fatty acids, but data were characterized by the reduced validity of the endpoints. In general, trial quality was found to be poor. Studies on vitamin supplementation were not analyzed as none met the eligibility criteria, mainly due to lack of clinical outcomes. Thus, evidence on the benefits and risks of vitamin supplementation and antioxidant supplements in MS is lacking.

## Suggestions for a Nutritional Intervention in MS: The Choice of Diet and Dietary Supplements

At the end, the goal of a nutritional intervention in MS must be the control of inflammation and this, as shown in this review, can be achieved mainly by controlling postprandial inflammation, the composition of gut microbiota and intestinal and systemic inflammation, and immunity. This can be achieved by a long-term dietary intervention, with a hypocaloric diet, prebiotics, probiotics, and dietary supplements.

As reported in this article, healthy dietary molecules, calorie restriction, and exercise are able to direct cell metabolism toward catabolism and downregulate anabolism and inflammation by interacting at different levels with specific enzymes, nuclear receptors, and transcriptional factors. Furthermore, in association with fiber, they can shift gut dysbiosis to eubiosis.

As a result, low-calorie meals (1,600–1,800 kcal) based on vegetables, whole cereals, legumes, fruit, and fish may slow down the progression of the disease and ameliorate the wellness of MS patients, whereas hypercaloric diets with high intake of salt, saturated animal fat, fried food, and sugar-sweetened drinks may lead to the onset of postprandial inflammation and systemic low-grade inflammation.

Diet should be integrated with prebiotics, probiotics, specific vitamins (D, A, B12, and nicotinic acid), oligoelements (magnesium and selenium), and dietary supplements such as polyphenols, n-3 PUFA, and lipoic acid.

Prebiotics for MS should include inulin, bran, lactosucrose, and oligofructose, preferential nutrients for colonocytes and capable to inactivate NF-kB. Probiotics, such as *lactococcus lactis*, *bifidobacterium lactis*, and *clostridium butyricum*, which can improve the intestinal microbial balance, can be used to change the composition of colonic microbiota. The combination of prebiotics and probiotics is highly recommended. Bowel functions and weight should always be under control.

A more drastic therapeutic approach aimed to restore gut eubiosis and downregulate inflammation may be represented by fecal microbiota transplantation (FMT; Smits et al., 2013). The method seems to be very effective but still primitive, not completely safe, and in a way also disgusting. The field should move beyond fecal transplants, identify the organisms that may be essential for a particular condition, and provide those organisms in a much simpler fashion than FMT (“Critical Views in Gastroenterology & Hepatology,” 2014).

Dietary supplements, with the only exception of omega-3 PUFA, which are normal constituents of our body, are useful at the beginning of the nutritional intervention, or in the course of relapses, to facilitate the recovery of a healthy condition, but their use should be restricted to only a limited period of time (3–4 months). This is particularly valid for the polyphenols. Polyphenols are not well-known molecules with regard to their bioavailability and their biological effects and special precautions should be used when supplementing the diet with them. On one hand, they can downregulate the synthesis of proinflammatory molecules in the course of inflammatory processes; on the other hand, they can stimulate cell activity in resting cells, but a persistent stimulation can induce the apoptosis of healthy cells. Taken together, these considerations suggest that administration of purified polyphenols should be performed on the basis of preliminary clinical trials to test their effectiveness as dietary supplements and to determine their long-term safety and the right dosage.

In general, a nutritional intervention with anti-inflammatory food and dietary supplements decreases the biosynthesis of proinflammatory compounds and therewith makes more effective the use of immune-modulatory drugs, and eventually might limit their possible adverse effects, alleviate the symptoms of chronic fatigue syndrome, and favor patient wellness. However, diet and dietary supplements should not be treated as drugs and as a substitute of therapy. Similarly, proinflammatory food is not toxic and there is no need to exclude it completely. You can eat a nice steak or fried food without risk or guilt, if you are in a basically healthy condition. What hurts are the wrong eating habits in the long run.

## Conclusions

So, at first glance, MS does not seem to have any of the characteristics of chronic inflammatory diseases, which could be related to wrong dietary habits and lifestyle, or even to a dysbiotic gut microbiota. There is apparently nothing in an exacerbation of the disease that may be linked to food or the state of the intestinal microbiota. In fact, when we began our studies on the impact of nutrition on MS, there was not even the slightest clue that there could exist a real link between them, and the idea of the involvement of gut microbiota in MS was considered only very speculative. To date, the idea that dietary habits might influence the course of MS is still struggling to establish itself. Not so in cardiovascular diseases and other chronic inflammatory conditions, in which the influence of dietary habits is almost accepted, and not even in cancer, which is increasingly considered as a metabolic disorder (Seyfried et al., 2014).

At present, MS therapy is not associated to any particular diet, probably due to lack of information on the effects of nutrition on the disease. However, the majority of patients with MS is looking for complementary and alternative treatments (CAM), and in particular is trying to change dietary habits, almost without the advice of the physician (Schwarz et al., 2008; Leong et al., 2009). A recent study based on data provided by MS patients in response to a questionnaire on their dietary habits seems to support a significant association of healthy dietary habits with better physical and mental health-related quality of life and a lower level of disability (Hadgkiss et al., 2014). These data reinforce the idea of the need for randomized controlled trials of nutritional intervention for people with MS. It should be emphasized that nutritional treatments should be complementary, but not alternative to therapy, be part of a holistic approach and performed under medical control.

As there are no data available from clinical trials yet, our work is aimed to rationalize dietary choices on the basis of known and established effects of dietary factors and lifestyle at the molecular level. Data reported in Figure 2 are obviously not complete but may be useful to provide guidelines for nutritional interventions. In principle, proinflammatory food upregulate the biosynthetic and inflammatory pathways, as shown on the right and at the bottom of Figure 2, whereas anti-inflammatory food upregulates oxidative metabolism and downregulates anabolism and inflammation.

As shown in this article, the finding that calorie restriction, exercise, and particular dietary factors can influence the degree of inflammatory responses by acting on both cellular metabolism (Figure 2) and composition of gut microbiota (Figure 5), suggests that an appropriate nutritional intervention may ameliorate the course of the disease and may be therefore taken in consideration as a possible complementary treatment in MS. As inflammation is present in both RRMS and PPMS, nutritional advices are indicated for both forms of the disease. This is particularly important in the case of PPMS, for which no cure is presently available. Conversely, as specific dietary habits may be detrimental and may promote a chronic state of low-grade inflammation, a wrong diet may be considered a possible contributory cause of relapses in MS.

Taken together, we have now a better knowledge of the possible influence of dietary factors on cell metabolism and gut microbiota, and on their possible effects on the disease, but, clearly, we are only just beginning to understand the role of nutrition and gut microbiota in MS and much work remains in terms of understanding the nature of the interactions of gut microbiota with the host’s immune system, especially at sites distal to the intestine.

On these grounds, future prospects in MS research should regard the following points: (a) assess gut microbiota composition; (b) evaluate defects in intestinal immune system; (c) clarify the role of polyphenols and vitamin D metabolism; (d) study the impact of dietary factors, herbs, and drugs on AMPK, Sirtuins, PPAR, or directly on NF-kB. Noteworthy, some drugs used to treat type II diabetes, such as the PPAR-γ agonists thiazolidinediones (Bernardo et al., 2009), and the AMPK agonist metformin (Nath et al., 2009) have anti-inflammatory effects comparable with those of anti-inflammatory dietary factors; (e) define possible interferences between dietary supplements and MS drugs; (f) promote a campaign aimed to educate about the importance to follow a healthy diet during therapy, for instance, encouraging patients to include fiber or complex carbohydrates in their diet, supplementing with probiotics, choosing n-3 fats over proinflammatory n-6 fats, and limiting meat and animal fat consumption. The choice of good recipes, such as those described by Mollie Katzen (2013), can make the diet more acceptable.

Overall, immune-modulatory conventional MS therapies have been almost successful; however, drugs that can protect and favor repair mechanisms are still missing. We can decide to help people stay healthy by providing nutritional guidance and physical activity opportunities. For the moment, there are only good prospects for improving the wellbeing of patients with MS. We are only at the beginning of the story.

## Summary

As both relapsing-remitting MS and primary-progressive MS are inflammatory diseases, they can be influenced by proinflammatory or anti-inflammatory dietary habits and lifestyle through their action on cell metabolism and gut microbiota. Nutritional advice to MS patients may favor their wellness.
